# Do Web-Based Interventions Improve Well-Being in Type 2 Diabetes? A Systematic Review and Meta-Analysis

**DOI:** 10.2196/jmir.5991

**Published:** 2016-10-21

**Authors:** Michelle Hadjiconstantinou, Jo Byrne, Danielle H Bodicoat, Noelle Robertson, Helen Eborall, Kamlesh Khunti, Melanie J Davies

**Affiliations:** ^1^ Diabetes Research Centre Department of Health Sciences University of Leicester Leicester United Kingdom; ^2^ Leicester Diabetes Centre NHS Trust University Hospitals of Leicester Leicester United Kingdom; ^3^ Diabetes Research Centre College of Medicine, Biological Sciences and Psychology University of Leicester Leicester United Kingdom; ^4^ School of Clinical Psychology University of Leicester Leicester United Kingdom; ^5^ Social Science Applied to Healthcare Improvement Research (SAPPHIRE) Group Department of Health Sciences University of Leicester Leicester United Kingdom

**Keywords:** type 2 diabetes, Web-based intervention, Internet, well-being, systematic review, meta-analysis

## Abstract

**Background:**

Poor diabetes self-care can have a negative impact on psychological well-being and quality of life. Given the scarcity of traditional psychological support and the barriers to uptake of and attendance at face-to-face education programs, Web-based interventions are becoming a popular approach to provide an additional platform for psychological support in long-term conditions. However, there is limited evidence to assess the effect of Web-based psychological support in people with type 2 diabetes.

**Objective:**

This systematic review is the first review to critically appraise and quantify the evidence on the effect of Web-based interventions that aim to improve well-being in people with type 2 diabetes.

**Methods:**

Searches were carried out in the following electronic databases: MEDLINE, EMBASE, CINAHL, PsycINFO, and Cochrane Library. Reference lists were hand-searched. A meta-analysis was conducted for depression and distress outcomes.

**Results:**

A total of 16 randomized controlled studies met the inclusion criteria for the systematic review and 9 were included in the meta-analyses. Theories were applied to the majority of the interventions. The most common behavior change techniques were “General information” and “Tracking/monitoring.” Interventions with a duration of 2-6 months providing professional-led support with asynchronous and synchronous communication appeared to be associated with significant well-being outcomes. The pooled mean (95% confidence interval) difference between the intervention and control arms at follow-up on depression score was -0.31 (-0.73 to 0.11). The pooled mean difference on distress scores at follow-up was -0.11 (-0.38 to 0.16). No significant improvements in depression (*P*=.15) or distress (*P*=.43) were found following meta-analyses.

**Conclusions:**

While the meta-analyses demonstrated nonsignificant results for depression and distress scores, this review has shown that there is a potential for Web-based interventions to improve well-being outcomes in type 2 diabetes. Further research is required to confirm the findings of this review.

## Introduction

Diabetes has become a global health concern, with 415 million people estimated to be living with diabetes worldwide. This figure is estimated to rise to around 642 million by 2040, with approximately 90% of those cases being type 2 diabetes mellitus [1-3]. Despite a growing number of treatment and therapy options available to people with type 2 diabetes, the number of diabetes-related complications continues to rise [4]. Risk of such complications can be reduced by making appropriate lifestyle changes in addition to diabetes therapies [5]. However, for some, making these changes can become overwhelming, as they must adjust to a new lifestyle and live with diabetes for the rest of their life [6-8]. National and international surveys highlight that poor diabetes self-care and the daily demands of diabetes management can lead to low quality of life and poor well-being [9-12]. The prevalence of poor psychological health is evident, with depression twice as common in people with type 2 diabetes, than those without the condition [13-16], and with distress affecting 10-30% of people with type 2 diabetes [17], leading to poor glycemic control, medication adherence, and overall low health outcomes [18-22].

### Well-Being

The Diabetes Management and Impact for Long-term Empowerment and Success report defines well-being as how satisfied an individual is with their quality of life. Other sources state that quality of life is not the end-all definition of well-being but is in fact one of many elements of well-being [23]. The World Health Organization defines well-being as when an individual “…can cope with the normal stresses in life, can work productively and is able to make a contribution to his/her community” [24], whereas the National Institute for Health and Care Excellence guidelines define well-being as when a person is happy and confident with no feelings of anxiety or depression, managing their feelings and emotions and being resilient [25].

It is evident that well-being remains a complex, multifaceted construct that is used interchangeably with various definitions existing across the literature demonstrating subjectively experienced domains and constructs [26,27]. The unclear definition of well-being creates difficulties in measuring this construct, and as a consequence, there are currently numerous questionnaires that measure a wide range of psychological constructs that include aspects of well-being, such as depression, distress, and quality of life [26].

### Web-Based Programs

Diabetes self-management education, including structured education and behavior change programs, can prevent or prolong diabetes-related complications [28,29]. However, there is a reported gap in these services’ provision of support focusing on well-being [10,30]. Attendance rates at self-management programs are reported to be low due to logistical or infrastructure issues that may contribute to low uptake [31,32]. Given the scarcity of psychological support provided through local services and the barriers to uptake at traditional education programs, Web-based interventions are becoming an additional or alternative provider of support to people with long-term conditions, including type 2 diabetes [33-36].

### Evidence on Web-Based Interventions in Type 2 Diabetes and Well-Being

Web-based interventions are described as self-guided programs that aim to change and improve knowledge and awareness around a health condition. Evidence indicates that such interventions are cost-effective, able to reach a wide range of audiences, especially those with a more restrictive lifestyle [37,38]. Recent reviews of Web-based interventions in type 2 diabetes have suggested positive impacts for outcomes of depression and anxiety [35,39]. Other studies and meta-analyses that looked at Web-based interventions for depression also reported effectiveness in elevating lowered mood [40,41]. Some recent reviews, however, have demonstrated no significant improvement in depression or distress [33,34]. Overall, current literature illustrates that there is limited evidence around the effect of such interventions on well-being in people with type 2 diabetes.

According to Corbin and Strauss, self-management programs, whether face-to-face or online, must consist of three constructs: medical, emotional, and role management. For example, they must include tasks around medical or diet adherence (medical self-management), tasks in changing or maintaining new behavioral/life roles within social relationships (role management), or tasks in coping with the emotional burden of living with a long-term condition (emotional management) [42]. To our knowledge, existing reviews mostly focus on medical management [43]. For instance, a recent review that explored online self-management interventions around lifestyle modification examined outcomes that were behavioral (role) and physiological (medical), excluding psychological and emotional management [44].

The aim of this paper is to report the first systematic review to identify and evaluate the current literature on Web-based programs or interventions for emotional management in type 2 diabetes and their impact on well-being.

## Methods

### Reporting Standards

This systematic review has been registered on PROSPERO (No. CRD42015020281) and meets the requirements of the Preferred Reporting Items for Systematic Reviews and Meta-Analyses (PRISMA) statement [45].

### Data Sources and Search Methods

The search strategy was carried out to identify relevant studies using the following five databases: MEDLINE, Embase, CINAHL, PsycINFO, and Cochrane Library.

Databases were searched using a combination of Medical Subject Headings (MeSH) topics and free-text terms. An example of the full electronic search strategy used for the databases is included in Multimedia Appendix 1. Publication year was between 1995 and 2016; this limitation was based on the recognition that the Internet became mainstream in 1995 with the launch of Windows 95; therefore, any use of the Internet prior to 1995 would not have met the study criteria [46].

After conducting the search, duplicates were removed and 2 reviewers (MH and JB) independently checked the titles and abstracts. The full text of the remaining papers was retrieved and again independently assessed for inclusion by the same 2 reviewers. Discrepancies were resolved through a third reviewer (DB). Reference lists of included papers were hand-searched. Experts in this topic area were contacted to ensure recent publications were included in this review.

### Study Selection

We included studies that were randomized controlled trials (RCTs), including RCT feasibility or pilot studies, with a follow-up of at least 2 months (8 weeks). Due to inconsistent usage of the term “well-being” across the literature and to ensure that no relevant papers were omitted and that the deficits and assets of the term “well-being” were captured, it was decided to use “well-being” as an umbrella term and include the following outcomes: well-being, distress, depression, anxiety, quality of life, self-efficacy, and social support. We incorporated studies that used validated tools to measure the above outcomes.

For the purpose of this review, Web-based interventions are defined as an intervention that may comprise modules or can be a health-related website that aims to change an outcome. Studies were included if they evaluated one Web-based/online intervention, with a combination of other modes, such as telephone calls or SMS (short message service) texts, that provided information, education, peer support, and/or overall therapeutic components to people with type 2 diabetes over the age of 18 years. Studies with any participants with type 2 diabetes (including studies with both type 1 diabetes and type 2 diabetes) were included in the review. Studies were excluded if they were computer-based and not Internet-based, such as studies using a computer for glucose monitoring. Also, studies were excluded if they were not RCTs and if they did not measure well-being or its constructs as a primary or secondary outcome.

### Data Extraction and Quality Assessment

An appropriate quality assessment tool was used to assess the validity of the methodology following the Centre for Reviews and Dissemination guidelines [47]. The quality appraisal checklist, the Jadad scale, is used to help assess the quality of the design and conduct of RCTs. The Jadad scale is a 7-item scale and consists of questions indicating whether the quality of the trial is good or poor. Despite the negative criticism of this scale around allocation concealment, this scale has a strong emphasis on the report of trials and was considered appropriate for the review of RCTs [48]. The quality assessment was carried out independently by 2 researchers (MH and JB). A third assessor was consulted in the case of a disagreement (DB).

A standardized data extraction form was used for this review. Qualitative information, including a summary of the interventions and results, was extracted separately. Two reviewers (MH and JB) independently extracted the data and discussed any discrepancies. Where data were missing for the meta-analysis, authors of the eligible studies were contacted.

### Data Synthesis

Due to the heterogeneity of the study designs, interventions, and outcomes, qualitative data were summarized and collated using a descriptive data synthesis. Due to the inconsistency of outcome measures across the studies, a meta-analysis was carried out for two outcomes (ie, depression and distress), as these outcomes were reported in the majority of the studies. Both depression and distress were treated as separate constructs. Measures that were used for depression and distress were validated and were as follows. For depression, we included studies that used questionnaires such as the Center for Epidemiologic Studies Depression Scale (CES-D), the Patient Health Questionnaire (PHQ-9), or the Hospital Anxiety and Depression Scale (HADS). For distress, we included studies that used questionnaires such as the Problem Areas in Diabetes Questionnaire (PAID), the Diabetes Distress scale (DDS), or the Health Distress Scale (HDS).

Pooled mean depression and distress scores were estimated separately using random-effects meta-analysis to account for the large heterogeneity that was observed. Standardized means were used to account for the different scales used to measure depression and distress. Publication bias was assessed using the Egger test, and heterogeneity was assessed using the I^2^statistic. There were insufficient data to allow subgroup analyses or meta-regression analyses to be performed.

Sensitivity analyses were performed by pooling means depression and distress scores: (1) excluding pilot/feasibility studies, and (2) excluding trials with type 1 diabetes and type 2 diabetes.

All analyses were performed in Stata version 14 (StataCorp), using the METAN command for continuous data.

## Results

### Study Selection

**Figure 1:**
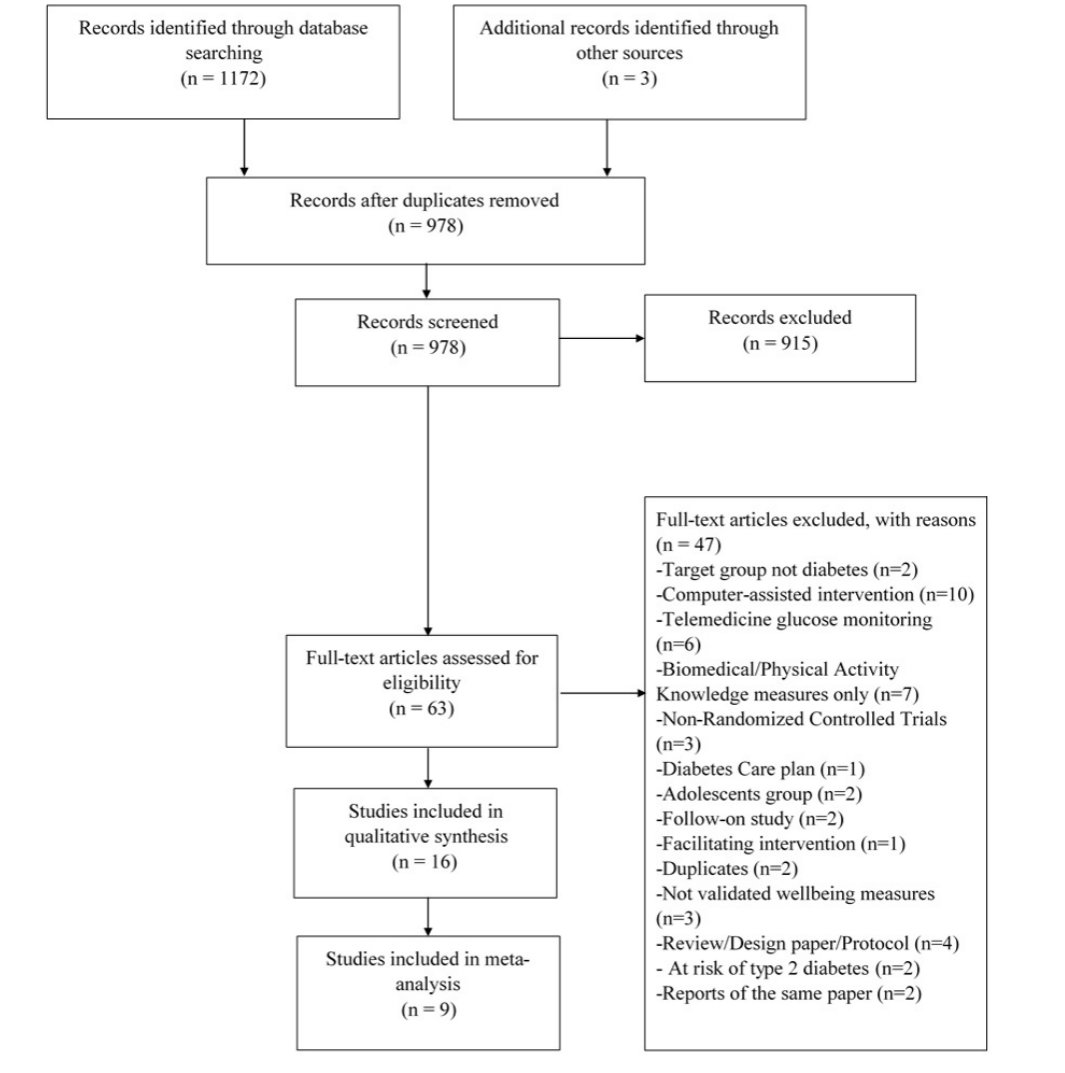
Study selection process.

The search identified 1172 potentially eligible articles (Figure 1). Of these, 63 full texts were assessed for eligibility. Figure 1 illustrates the main reasons for exclusion of articles. Three papers by different first authors reported the same study with identical study population [49-51]; therefore, only one paper was included in the review [50]. A total of 16 studies met the predefined criteria and were included in the review [50,52-66] (Figure 1).

### Study Characteristics

A total of 15 studies used a parallel RCT design, with one study using a crossover design [52]. We identified 14 studies that recruited patients diagnosed with type 2 diabetes [50,52,54-65], while four out of those studies recruited participants with both type 2 diabetes and type 1 diabetes [60,61,63,64]. Two studies did not specify the type of diabetes [53,66]. Studies were reported between 2002 and 2015 and were predominantly based in the United States (n=12), with one study carried out in each of the following countries: Canada [65], Norway [63], Germany [60], and the Netherlands [61]. The total number of participants across the studies was 3612 with a range of 17-761 (mean 220.32, SD 172.15). All 16 studies recruited more women (2208/3612, 61.13%) than men (1404/3612, 38.87%). The mean age across the studies was 53.4 years (range 23.9-67.2 years). Seven out of 16 studies (47%) reported having a predominantly white population (Table 1). A total of six studies (40%) did not report the ethnicity of their study population [57,58,62-64]. The interventions and control groups are described in Multimedia Appendix 2.

**Table 1:** Characteristics of the studies included in the review.

Study (year) and location	Name of intervention	Well-being outcome	Type of diabetes	Duration of intervention, months	Total N	Usage over time
Bond (2010) USA [53]	—	Depression Self-efficacy Quality of life Social support	Not specified	6	62	Not reported
Tang (2013) USA [54]	EMPOWER-D	Depression Distress	Type 2	12	415	Not reported
Heisler (2014) USA [55]	iDecide	Distress Self-efficacy	Type 2	3	188	Not reported
Glasgow (2012) USA [56]	CASM	Quality of life Self-efficacy	Type 2	12	463	Declined
McMahon (2012) USA [57]	—	Distress	Type 2	12	151	
McKay (2001) USA [50]	D-Net	Depression	Type 2	2	78	Declined
McKay (2002) USA [58]	D-Net	Depression Quality of life	Type 2	3	160	Declined
Lorig (2010) USA [59]	IDSMP	Depression Distress Self-efficacy	Type 2	6-18	761	Not reported
Nobis (2015) Germany [60]	GET.ON Mood	Depression Distress	Both types 1 and 2 (76% T2D^a^)	2	260	—
Van Bastelaar (2011) Netherlands [61]	—	Depression Distress	Both types 1 and 2 (82% T2D)	2	255	Not reported
Fisher (2013) USA [62]	REDEEM (CASM)	Distress	Type 2	12	392	Not reported
Wangberg (2008) Norway [63]	—	Self-efficacy	Both types 1 and 2 (28% T2D)	1	64	—
Hunt (2014) USA [52]	—	Self-efficacy	Type 2	3	17	Not reported
Smith (2000) USA [64]	Women to Women	Quality of life Social Support	Both types 1 and 2 (80% T2D)	5	30	Declined
Pacaud (2012) Canada [65]	—	Quality of life Self-efficacy	Type 2	12	68	Not reported
Fonda (2009) USA [66]	MyCare Team	Distress	Not specified	12	104	Not reported

^a^T2D: type 2 diabetes

**Table 2:** Methodological quality assessment per intervention.

Study (year)	Criteria							
	Eligibility criteria	Method of randomization	Single-blinded	Description of intervention	Description of withdrawals	Timing of assessment	Sample size calculation	Intention-to-treat analysis
Bond (2010) [53]	✓	✓	✓	✓	x	✓	✓	x
Tang (2013) [54]	✓	✓	✓	✓	✓	x	✓	✓
Heisler (2014) [55]	x	✓	x	✓	✓	✓	✓	x
Glasgow (2012) [56]	✓	✓	x	✓	✓	✓	✓	✓
McMahon (2012) [57]	✓	✓	x	✓	✓	✓	x	✓
McKay (2001) [50]	✓	✓	x	✓	✓	✓	x	x
McKay (2002) [58]	✓	x	x	✓	✓	✓	x	x
Lorig (2010) [59]	✓	✓	✓	✓	✓	✓	✓	x
Nobis (2015) [60]	✓	✓	x	✓	✓	✓	✓	x
Van Bastelaar (2011) [61]	✓	✓	✓	✓	✓	✓	✓	✓
Fisher (2013) [62]	✓	✓	✓	✓	✓	✓	x	x
Wangberg (2008) [63]	✓	x	x	✓	x	x	✓	x
Hunt (2014) [52]	✓	✓	x	✓	✓	✓	x	x
Smith (2000) [64]	✓	✓	x	✓	✓	✓	x	x
Pacaud (2012) [65]	x	x	x	x	x	✓	x	✓
Fonda (2009) [66]	x	x	x	✓	x	✓	x	x

### Methodological Quality Assessment

The methodological quality of the studies was generally high (Table 2). Nevertheless, some aspects, such as intention-to-treat, single-blinding, and sample size calculation, were not clearly reported in some studies.

#### Descriptive Data Synthesis

The most common duration of the interventions was 12 months [54,56,57,62,65,66]. Compliance rates ranged between 42-100%, while attrition rates were reported by the majority of the studies (n=13); these ranged from 6-22%. A few studies (n=4) reported a decline of intervention adherence over time [50,56,58,64]; reporting that the usage declined over 8 weeks [50], 5 months [64], and 12 months [56] (Table 1).

#### Modes of Communication and Type of Intervention Providers

The communication between intervention provider and/or peers was synchronous (eg, telephone calls) and/or asynchronous (eg, bulletin boards). Intervention providers were those involved in running the online intervention and often had direct or indirect contact with the users. They varied across the studies as follows: psychologists (n=4), nurses (n=6), dieticians (n=3), diabetes educators (n=2), coaches (n=2), social worker (n=1), physician (n=1), pharmacist (n=1), and endocrinologist (n=1). Two studies included nonprofessional providers [59,62], such as lay people and graduates, whereas three studies failed to report any characteristics of their intervention providers [55,63,65].

10 studies provided both asynchronous and synchronous communication [53,56-60,62,64-66], whereas six studies provided communication both with providers and other users [50,53,56,59,64,65]. Out of the seven studies that provided peer support, four were moderated [56,58,59,64], one was not moderated [53], and two studies did not report on moderation [50,65]. The intervention modules varied between 6-8 sessions. Half the studies specified the duration of their modules (which were online sessions); these varied from 45-120 minutes [54-56,59-62,65].

#### Theories and Behavior Change Techniques

Six studies failed to report whether their intervention was theory based [42,53,57,64-66]. The remaining ten studies were based on at least one theory: the Chronic Care Model [54], Motivational Interviewing [55,62], Social Cognitive Theory [56,63], Social Ecological Model [50,56], Self-Efficacy Theory [58], Social Support Theory [51], Systematic Behavioral Activation [60], Cognitive Behavioral Theory [61], or Self-Determination Theory [52].

All studies explicitly reported at least one behavior change technique, which we attempted to map onto Michie’s taxonomy [67] as follows: information provision (n=14); tracking/self-monitoring (n=12); providing motivation (n=12); providing feedback (n=9); goal setting (n=9); problem solving (n=9); action planning (n=7); social support (n=7); emotional control training (n=6); and prompt review of behavioral goals (n=1) (Table 3).

**Table 3:** Behavior change techniques used in interventions.

Study (year)	Behavior change techniques									
	General information	Goal setting	Action planning	Problem solving/ barrier	Prompt review of behavioral goals	Prompt self-monitoring/ tracking	Social support	Emotional control training	Motivational approach	Provide feedback on performance
Bond (2010) [53]	✓	✓	✓	✓	x	✓	✓	✓	✓	x
Tang (2013) [54]	✓	✓	✓	x	x	x	x	✓	✓	x
Heisler (2014) [55]	✓	✓	✓	✓	x	✓	x	x	✓	x
Glasgow (2012) [56]	✓	✓	✓	✓	x	✓	✓	x	✓	✓
McMahon (2012) [57]	✓	x	x	x	✓	✓	x	x	✓	✓
McKay (2001) [50]	x	✓	✓	✓	x	✓	✓	x	✓	✓
McKay (2002) [58]	✓	✓	x	✓	x	✓	✓	✓	✓	✓
Lorig (2010) [59]	✓	x	✓	✓	x	✓	✓	✓	✓	x
Nobis (2015) [60]	✓	✓	x	✓	x	x	x	✓	✓	✓
Van Bastelaar (2011) [61]	✓	x	x	x	x	x	x	x	x	✓
Fisher (2013) [62]	x	✓	✓	✓	x	✓	x	x	✓	✓
Wangberg (2008) [63]	✓	x	x	x	x	x	x	x	x	✓
Hunt (2014) [52]	x	x	x	x	x	✓	x	x	x	x
Smith (2000) [64]	✓	x	x	x	x	x	✓	x	x	x
Pacaud (2012) [65]	✓	x	x	x	x	✓	✓	x	x	x
Fonda (2009) [66]	✓	x	x	x	x	✓	x	x	x	x

**Table 4:** Primary targets and outcomes (primary or secondary) for each intervention.

Study (year)	Primary target	Outcome				
		Depression	Distress	Quality of life	Self-efficacy	Social support
Bond (2010) [53]	Psychosocial well-being	Primary		Primary	Primary	Primary
Tang (2013) [54]	Disease management	Secondary	Secondary			
Heisler (2014) [55]	Unspecified		Secondary		Secondary	
Glasgow (2012) [56]	Psychosocial outcomes			Primary	Primary	
McMahon (2012) [57]	Diabetes-related outcomes		Secondary			
McKay (2001) [50]	Physical activity levels	Primary				
McKay (2002) [58]	Unspecified	Primary		Primary		
Lorig (2010) [59]	HbA_1c_, exercise, self-efficacy, patient activation	Secondary	Secondary		Secondary	
Nobis (2015) [60]	Depression	Primary	Secondary			
Van Bastelaar (2011) [61]	Depression	Primary	Secondary			
Fisher (2013) [62]	Diabetes distress, self-management		Primary			
Wangberg (2008) [63]	Diabetes self-care behaviors				Secondary	
Hunt (2014) [52]	Self-efficacy, self-management, diabetes outcomes				Primary	
Smith (2000) [64]	Attitudes			Primary		Primary
Pacaud (2012) [65]	Unspecified			Secondary	Secondary	
Fonda (2009) [66]	Diabetes distress		Primary			

#### Outcomes and Measures

There was a variety of questionnaires used across studies to measure the same outcome. For depression, the following measures were used: CES-D [50,53,58,60,61], PHQ-9 [54,59], and HADS [60]. For distress, studies used PAID [54,57,60,61,66], DDS [55,62], and HDS [59]. Quality of life was assessed by using PAID [53], DDS [56], the Short Form-12 (SF-12) [58], the Quality of Life Index (QoL Index) [64], and the Diabetes Quality of Life Questionnaire [65]. Social support was assessed using the Diabetes Support Scale [53] and the Personal Resource Question [64], whereas self-efficacy was assessed by using the Diabetes Empowerment Scale [53], the Diabetes Self-Efficacy Scale [56,59], the Perceived Competence Scale [63], the Diabetes Management Self-Efficacy Scale [52], and the Rosenberg Self-Esteem Scale [65].

#### Improvements in Outcomes

Outcomes were measured as primary and/or secondary across the studies (Table 4). Five studies reported significant improvements in distress [55,60-62,66]. Three studies reported nonsignificant/significant improvements in depression [53,60,61]. Self-efficacy improved in four studies [53,56,59,65]. Quality of life showed some or little improvement in the majority of the studies [53,56,58,64]. Social support was significantly improved in one study [53] and “positively influenced” in another study [64].

A subset of the studies that had significant improvement in distress or depression shared some common characteristics [53,59-61]; that is, the interventions combined synchronous and asynchronous communication, with the intervention running between 2 and 6 months. Providers were mostly psychologists, and studies including peer support were moderated. General information was the most common behavior change technique.

### Meta-Analysis

Pairwise meta-analysis was carried out on a total of nine studies, with five studies included for depression scores only [50,53,58-60], six for distress scores only [54,55,57,59,60,62], and two studies analyzed for both outcomes [59,60]. The remaining seven studies from the qualitative data synthesis were excluded from the meta-analysis as there were not enough data to analyze each outcome.

#### Depression

**Figure 2:**
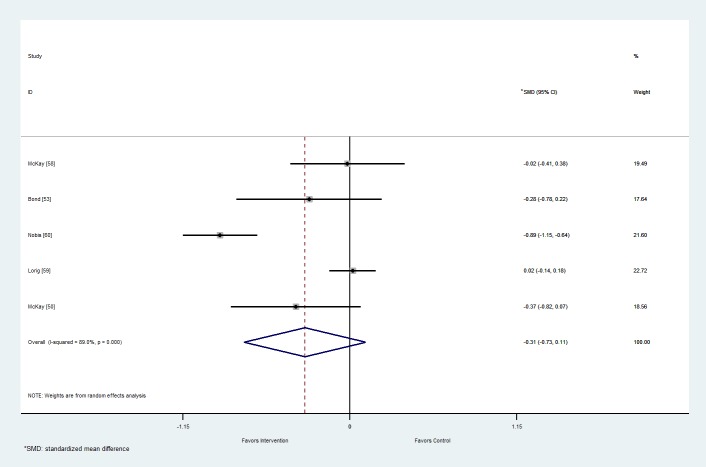
Forest plot of mean difference in depression score between the intervention and control arms at follow-up for studies including Web-based interventions and participants with type 2 diabetes mellitus. SMD: standardized mean difference.

From the five studies with outcome data for depression, the pooled mean (95% confidence interval) difference between the intervention and control arms on depression score was -0.31 (-0.73 to 0.11; Figure 2). The effect was not significant (*P*=.15). There was considerable heterogeneity (I^2^= 89%, *P*<.001). The funnel plot (Multimedia Appendix 3) and Egger’s test (*P*=.60) show no obvious publication bias.

#### Distress

**Figure 3:**
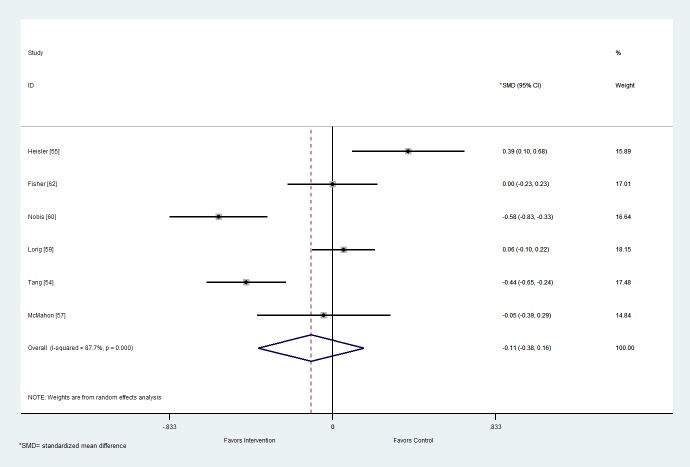
Forest plot of mean difference in distress score between the intervention and control arms at follow-up for studies including Web-based interventions and participants with type 2 diabetes mellitus. SMD: standardized mean difference.

From the six studies that reported outcome data for distress, the pooled mean (95% confidence interval) difference between the intervention and control arms on distress scores was -0.11 (-0.38 to 0.16; Figure 3). This effect was not significant (*P*=.43). There was considerable heterogeneity (I^2^=87.7%, *P*<.001). Egger’s test (*P=*.98) showed some indication of publication bias, but the funnel plot (Multimedia Appendix 4) suggests that some of the studies with a small negative standardized mean difference have not been reported. This suggests that the pooled mean may have been biased towards studies showing no effect or that control is preferable to intervention.

### Sensitivity Analyses

When studies with type 1 diabetes and type 2 diabetes participants were excluded for the outcomes depression and distress, the effect size was attenuated and was close to zero (Table 5). Excluding feasibility/pilot studies did not affect the main results (Table 5).

**Table 5:** Supporting table of pooled values.

Analysis	Depression	Distress
Main analyses	-0.31 (-0.73 to 0.11)	-0.11 (-0.38 to -0.16)
Without feasibility/pilot studies	-0.30 (-0.80 to 0.21)	-0.11 (-0.38 to -0.16)
Without T1D^a^ and T2D^b^ studies	-0.05 (-0.24 to 0.14)	-0.02 (-0.28 to 0.24)

^a^T1D: type 1 diabetes

^b^T2D: type 2 diabetes

## Discussion

### Principal Findings

To our knowledge, this is the first systematic review exploring solely the emotional management construct, specifically the following selected well-being elements: depression, distress, self-efficacy, quality of life, and social support. Individually, a number of studies obtained significant improvements in well-being measures. This improvement was not supported by the meta-analysis for the outcomes of depression and distress, confirming previous findings that Web-based interventions have little effect on distress [34] and emotional outcomes overall [33].

### Theories and Behavior Change Techniques

Unlike previous reviews on self-management Web-based interventions in type 2 diabetes [33], our review identified a number of theories across the majority of the papers. Evidence indicates that theory-based Web-based interventions are more effective [36] than non‒theory-based interventions [67,68]; however, there were no conclusive results regarding which theory was associated with the most improved outcomes. Theory-based interventions can help identify behavior change strategies that are also an important element during the development of a condition-specific intervention. In this case, we concluded that Web-based interventions included activities informed by behavior change techniques, with information provision and tracking as the most common techniques. It was evident that there was a wide range of common behavior change techniques used by the majority of the studies, resulting in an inability to identify which behavior change techniques are primarily used, and which are the most effective for this type of intervention. A similar result found in previous reviews on self-management type 2 diabetes interventions [33,36,69].

### Type of Intervention Providers

The current evidence around mental health support and online interventions remains divided, with some studies supporting that a professional-led intervention can be beneficial [40], while others suggesting that a non‒professional-led intervention can perform equally well [41,70]. In this review, the majority of studies that provided professional support showed more promising results than those providing nonprofessional support. This conclusion may be influenced by variation in the roles that these providers had in each study, but also the fact that the ratio of professional- and non‒professional-led support was uneven across each intervention, with the majority of the studies including professional-led support.

### The Need for Shared Definitions

Issues defining “well-being” and its constructs were iterated in our review. For example, one study [54] that stated it was exploring the well-being outcome, in fact did not assess well-being, nor did it use a well-being measure. Instead, the study measured the constructs “depression” and “distress” with depression- and distress-specific scales. Despite depression being considered as a more established construct and being separate to the construct distress, current literature has argued that both depression and distress are still being used interchangeably [17,71]. Depression and distress are both real established constructs, and even though they may overlap with one another, it is important that they be assessed independently.

### The Use of Appropriate Specific Outcome Measures

Another issue is the use of incorrect measures for specific outcomes. With distress becoming an established construct [17], it can be measured with validated and reliable distress questionnaires. Specifically, the DDS and PAID measures are both appropriate tools to assess and quantify the construct of distress. However, despite having existing validated measures for this specific construct, it appeared that some questionnaires were used for other outcomes. For example, in two studies, PAID and DDS scales (both distress measures) were used to measure quality of life. Incorrect use of outcome-specific measures can create barriers to distinguishing aspects of well-being.

### Strengths and Limitations

This review has used a robust search strategy, which identified a satisfactory number of studies and is reported in accordance with PRISMA guidelines [72] to determine the usefulness of such interventions for this patient group and to highlight key recommendations for future research in this area. The search was conducted on multiple electronic databases, reference lists were hand-searched, and experts in the area were contacted. The review was based on a strict inclusion and exclusion criteria, and 2 independent authors reviewed quality check and potential articles, and extracted data. Studies with participants with both type 1 diabetes and type 2 diabetes were considered in order to include people with type 2 diabetes and to be consistent with previous reviews. To ensure that the effect of changes was examined, sensitivity analyses were carried out excluding studies with participants with type 1 diabetes and type 2 diabetes. Sensitivity analyses were also carried out to exclude pilot/feasibility studies. Both sensitivity analyses further suggested that Web-based interventions demonstrate little improvement in depression and distress.

As with all systematic reviews, there are some limitations to consider. At a study level, the number of studies included in the meta-analysis was low, and there was considerable heterogeneity across studies with regard to intervention design and measurement of outcomes. This could relate to the fact that the primary aim within interventions varied, with some studies focusing on medical management tasks and other studies focusing on emotional management tasks. At a review level, especially when determining what studies should be included, the terms “well-being” and “Web-based interventions” were based on an in-depth review of the literature and in-depth discussions between 2 independent reviewers throughout the process. The lack of comparable data across all outcomes also led to a less reliable descriptive data synthesis being performed rather than a more robust meta-analysis; therefore, any conclusions must be considered with caution. To minimize bias, this review attempted to explain the results in a logical way for each of the included studies.

### Implications

Multicomponent interventions may be useful and may seem effective in studies (eg, Web-based, phone-based), but this creates a challenge for researchers to identify whether the intervention as a whole or only certain aspects contribute to the effect of the intervention. Several implications for the conduct of research in this area can be considered.

- Future RCTs looking at similar outcomes should consider using a similar approach to study and/or intervention design in order to make the comparison between interventions much easier, avoiding bias, and in essence producing more reliable conclusions; for example, robust reporting data in line with the Consolidated Standards of Reporting Trials (CONSORT) guidelines and measuring outcomes with similar questionnaires.
- Further research may be needed to examine the effect of Web-based interventions in well-being for people with type 2 diabetes, including long-term studies with larger sample sizes.
- Future studies may provide a full and detailed description of the intervention including its components to help determine why some studies have some effect and other studies have little or no effect on their outcome.
- The majority of RCTs measure psychological outcomes as secondary outcomes, focusing less on the emotional management tasks and more on the medical management. Future studies may aim to approach self-management interventions in a more holistic approach including all three constructs (medical, role, emotional) equally.
- Further research may require more consistent definitions of “well-being” and its constructs and may require consistent validated specific measures for each outcome.
- Michie’s Taxonomy of Behavior Change Techniques could be considered as a guide for a robust classification system.

### Conclusion

The findings of this review collated information and highlighted key issues with the evaluation of Web-based interventions for promoting well-being in people with type 2 diabetes (see Multimedia Appendix 5 for a summary of key findings). It has proposed some recommendations for future research to develop effective interventions. Such interventions could allow stakeholders and health care providers to provide effective, integrated, ongoing Web-based support to promote valuable emotional and general management of type 2 diabetes. Web-based interventions could supplement traditional face-to-face support to improve reach and sustainability and in turn create a more holistic approach to diabetes self-management, bridging the gap between diabetes support and diabetes self-care.

## Appendix

Multimedia Appendix 1 Search strategy.

Multimedia Appendix 2 Description of intervention and control groups per study.

Multimedia Appendix 3 Funnel plot for depression.

Multimedia Appendix 4 Funnel plot for distress.

Multimedia Appendix 5 Summary of key findings.

## Abbreviations

CES-D: Center for Epidemiologic Studies Depression Scale
CONSORT: Consolidated Standards of Reporting Trials
DDS: Diabetes Distress Scale
HADS: Hospital Anxiety and Depression Scale
HDS: Health Distress Scale
MeSH: Medical Subject Headings
PAID: Problem Areas in Diabetes Questionnaire
PHQ-9: Patient Health Questionnaire
PRISMA: Preferred Reporting Items for Systematic Reviews and Meta-Analyses
QoL Index: Quality of Life Index
RCT: randomized controlled trial
SF-12: Short Form-12
SMS: short message service
WHO: World Health Organization
