# Supplementary material for: Do Web-Based Interventions Improve Well-Being in Type 2 Diabetes? A Systematic Review and Meta-Analysis
Source: J Med Internet Res. 2016 Oct 21;18(10):e270. doi: 10.2196/jmir.5991 (PMC5097175; doi:10.2196/jmir.5991)
Supplement: Multimedia Appendix 1 [file jmir_v18i10e270_app1.pdf]

APPENDIX 1

Database: Ovid MEDLINE(R) In-Process & Other Non-Indexed Citations and Ovid MEDLINE(R) <1946 to Present>
Search Strategy:
----
1 exp diabetes mellitus, Type 2/ (94839)
2 (diabet* adj3 type adj "2").ab,ti. (4255)
3 T2DM.ab,ti. (8768)
4 T2D.ab,ti. (3691)
5 NIDDM.ab,ti. (6859)
6 (diabet* adj3 type adj ii).ab,ti. (945)
7 (non-insulin-dependent adj2 diabet*).ab,ti. (10016)
8 (adult-onset adj2 diabet*).ab,ti. (518)
9 1 or 2 or 3 or 4 or 5 or 6 or 7 or 8 (103718)
10 (computer-assist* or computer-based or web-based or online-based).mp. [mp=title, abstract, original title, name of substance word, subject heading word, keyword heading word, protocol supplementary concept word, rare disease supplementary concept word, unique identifier] (278055)
11 (computer* or telecommunication*).mp. [mp=title, abstract, original title, name of substance word, subject heading word, keyword heading word, protocol supplementary concept word, rare disease supplementary concept word, unique identifier] (654788)
12 (interactive or online or on-line or telemedicin*).mp. [mp=title, abstract, original title, name of substance word, subject heading word, keyword heading word, protocol supplementary concept word, rare disease supplementary concept word, unique identifier] (120421)
13 (world wide web or worldwide web or website*).mp. [mp=title, abstract, original title, name of substance word, subject heading word, keyword heading word, protocol supplementary concept word, rare disease supplementary concept word, unique identifier] (16772)
14 electronic health*.mp. (13124)
15 e-health.mp. (1373)
16 telehealth.mp. (2074)
17 Internet/ or internet.mp. (71746)
18 web*.mp. (86911)
19 technol*.mp. (352646)
20 10 or 11 or 12 or 13 or 14 or 15 or 16 or 17 or 18 or 19 (1144579)
21 9 and 20 (3201)
22 limit 21 to (humans and yr="1995 -Current" and randomized controlled trial and last 20 years) (376)
