# Supplementary material for: Do Web-Based Interventions Improve Well-Being in Type 2 Diabetes? A Systematic Review and Meta-Analysis
Source: J Med Internet Res. 2016 Oct 21;18(10):e270. doi: 10.2196/jmir.5991 (PMC5097175; doi:10.2196/jmir.5991)
Supplement: Multimedia Appendix 2 [file jmir_v18i10e270_app2.pdf]

Appendix 4: Description of intervention and control groups per study

First author (year)	Conditions/Assessments
**Bond (2010)**	T1: web-based interventions + usual care<br>C: Usual care
**Tang (2013)**	T1: PHCP (online disease management system) – glucometer and patient dashboard<br>C: NR
**Heisler (2014)**	T1: 1- to 2- hour sessions with a community health worker (iDecide)<br>C: 1- to 2- hour sessions with a community health worker (hardcopy)
**Glasgow (2012)**	T1:Internet based computer assisted self-management (CASM)<br>T2: CASM + enhanced social support<br>C: Enhanced usual care, computer-based feedback + recommendations minus key intervention procedures.
**McMahon (2012)**	T1: Online care management with asynchronous online communication and data sharing.<br>T2:Telephone- based care<br>T3: Usual care with computer use and internet access.<br>C: N/A
**McKay (2001)**	T1: eight week. D-Net Active Lives PA intervention<br>C: eight week. Internet-Information only. Access to diabetes-related articles in the web-site library. BG tracking
**McKay (2002)**	T1: Internet-based peer support<br>T2: Personalised self-management coach<br>T3: Combined condition<br>C: Information only
**Lorig (2010)**	T1: Program (six weekly sessions)<br>T2: Program + email reinforcement<br>C: Usual care
**Nobis (2015)**	T1: six consecutive sessions: guided self-help brief online unguided psycho-education intervention to reduce depression<br>C: Access to an online psychoeducational program, based on the German S3-Guidance/National Disease Management Guideline for Unipolar Depression.
**Van Bastelaar (2011)**	T1: eight consecutive lessons (written/verbal information)<br>C: 12 week waiting list. Completed measurements 8+12 weeks after randomisation. After waiting period, they had access to intervention.
**Fisher (2013)**	T1: 40min validated Internet-based computer assisted self-management program (CASM)<br>T2: CASM + PST (CAPS)<br>C: Computer minimal support
**Wangberg (2008)**	T1: Online intervention in self-care with low SE (LSE)<br>T2: Online intervention in self-care with high SE (HSE)
**Hunt (2014)**	T1: iPad (app called ‘Diabetes Buddy’)<br>C: journal
**Smith (2000)**	T1: 5 month computer-intervention (software First Class)<br>C: No computer
**Pacaud (2012)**	T1: Web static (electronic education, virtual appointments with asynchronous communication)<br>T2: Web interactive (electronic education, virtual appointments with asynchronous/synchronous communication)<br>C: Face-to-face education, synchronous/asynchronous communication.
**Fonda (2009)**	T1: 12 month web-based

	C: Usual care
