# Supplementary figures and images for: Do Web-Based Interventions Improve Well-Being in Type 2 Diabetes? A Systematic Review and Meta-Analysis
Source: J Med Internet Res. 2016 Oct 21;18(10):e270. doi: 10.2196/jmir.5991 (PMC5097175; doi:10.2196/jmir.5991)

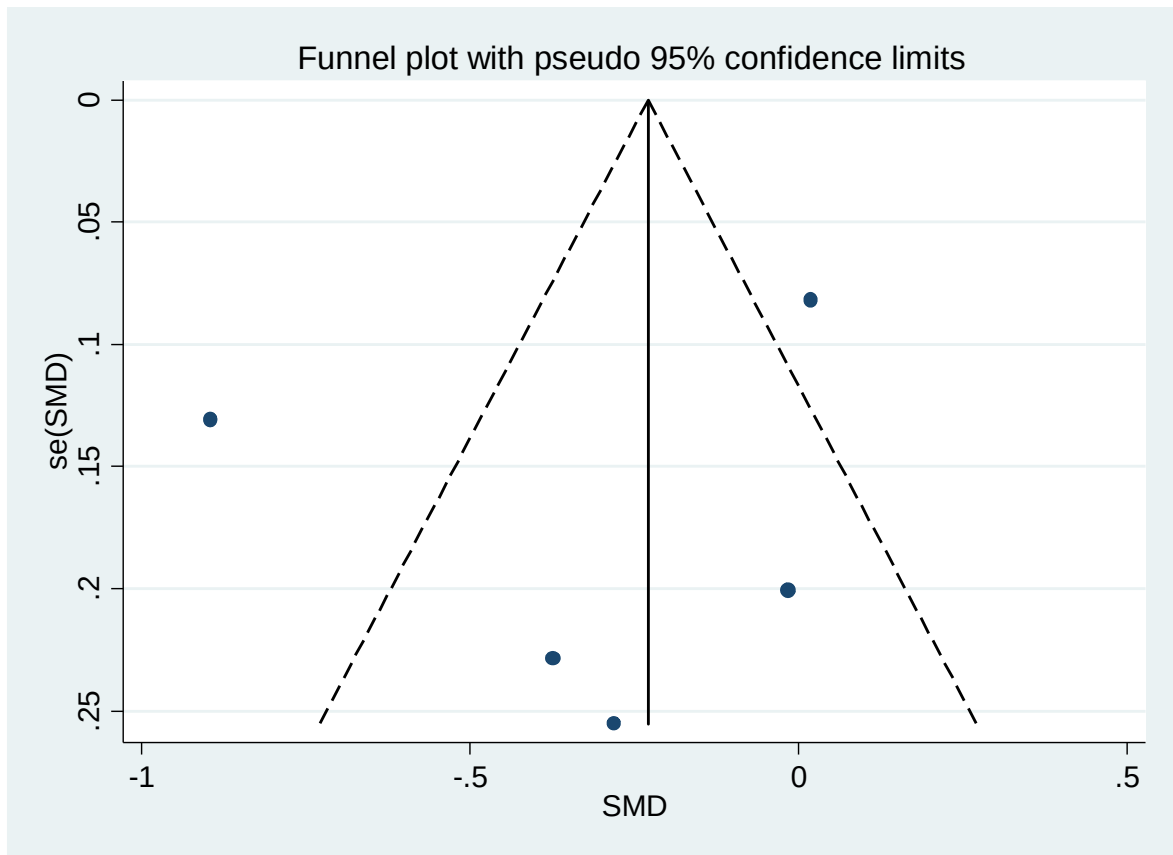

Multimedia Appendix 2: Funnel plot for depression

Supplement: Multimedia Appendix 3 [file jmir_v18i10e270_app3.pdf]

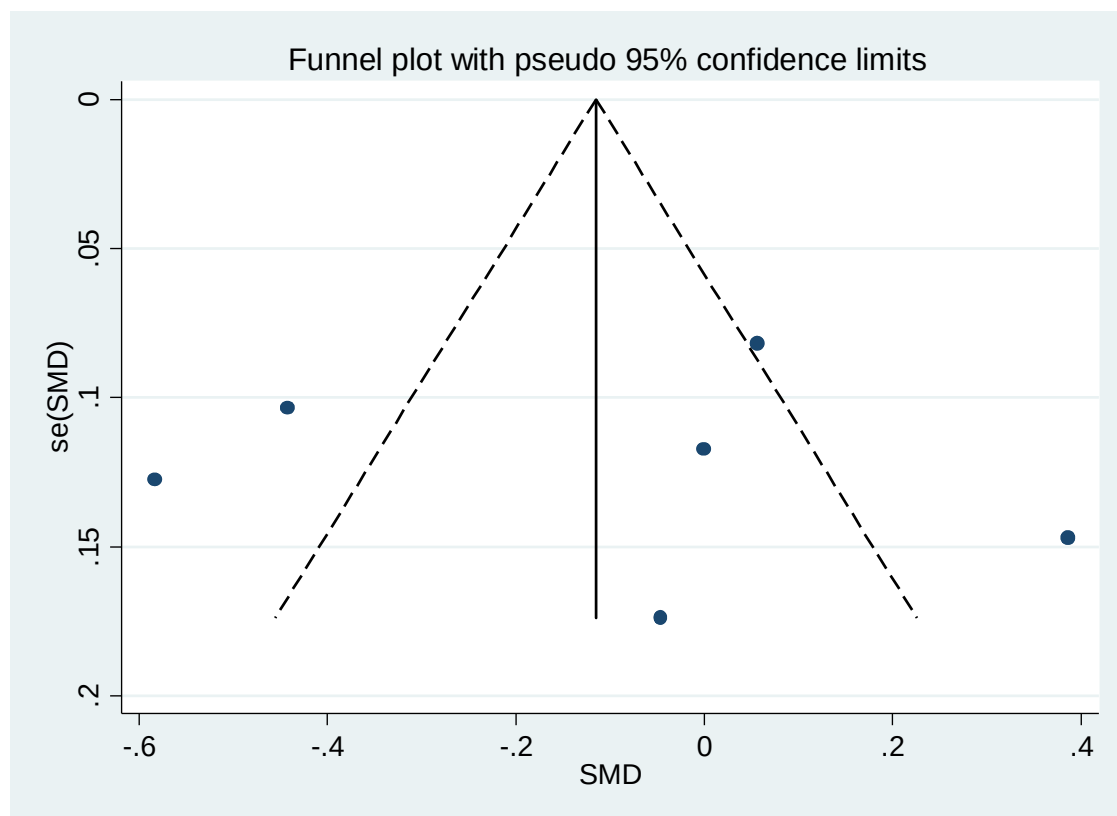

Multimedia Appendix 3: Funnel plot for distress

Supplement: Multimedia Appendix 4 [file jmir_v18i10e270_app4.pdf]
