# Supplementary material for: Do Web-Based Interventions Improve Well-Being in Type 2 Diabetes? A Systematic Review and Meta-Analysis
Source: J Med Internet Res. 2016 Oct 21;18(10):e270. doi: 10.2196/jmir.5991 (PMC5097175; doi:10.2196/jmir.5991)
Supplement: Multimedia Appendix 5 [file jmir_v18i10e270_app5.pdf]

Box 1: Summary of key findings

Summary of key findings:

- 16 studies met the criteria for review
- Methodological quality assessment of studies was generally well reported
- The majority of the studies were long term (>6 months). Those running between two to six months were associated with positive well-being outcomes.
- Most studies were based on a theoretical framework and involved multi components to engage and motivate participants to change behaviour
- ‘Information’ and ‘tracking’ were some of the most common behaviour change techniques applied in web-based interventions
- Professional-led online support was linked with positive well-being outcomes compared to those with non-professional-led support
- A combination of synchronous and asynchronous communication was linked with positive well-being outcomes.
